# Comparison of miRNAs and Their Targets in Seed Development between Two Maize Inbred Lines by High-Throughput Sequencing and Degradome Analysis

**DOI:** 10.1371/journal.pone.0159810

**Published:** 2016-07-27

**Authors:** Feng-Yao Wu, Cheng-Yi Tang, Yu-Min Guo, Min-Kai Yang, Rong-Wu Yang, Gui-Hua Lu, Yong-Hua Yang

**Affiliations:** 1 State Key Laboratory of Pharmaceutical Biotechnology, NJU-NJFU Joint Institute of Plant Molecular Biology, School of Life Sciences, Nanjing University, Nanjing 210093, China; 2 Co-Innovation Center for Sustainable Forestry in Southern China, Nanjing Forestry University, Nanjing 210037, China; Montana State University Bozeman, UNITED STATES

## Abstract

MicroRNAs (miRNAs) play an important role in plant growth, development, and response to environment. For identifying and comparing miRNAs and their targets in seed development between two maize inbred lines (i.e. PH6WC and PH4CV), two sRNAs and two degradome libraries were constructed. Through high-throughput sequencing and miRNA identification, 55 conserved and 24 novel unique miRNA sequences were identified in two sRNA libraries; moreover, through degradome sequencing and analysis, 137 target transcripts corresponding to 38 unique miRNA sequences were identified in two degradome libraries. Subsequently, 16 significantly differentially expressed miRNA sequences were verified by qRT-PCR, in which 9 verified sequences obviously target 30 transcripts mainly involved with regulation in flowering and development in embryo. Therefore, the results suggested that some miRNAs (e.g. miR156, miR171, miR396 and miR444) related reproductive development might differentially express in seed development between the PH6WC and PH4CV maize inbred lines in this present study.

## Introduction

MicroRNAs (miRNAs) are a class of endogenous, small RNAs (21–24 nt) that regulate gene expression in plants and animals at the post-transcriptional level by translational repression, target degradation and gene silencing [1–7]. Plant miRNAs play an important role in various processes associated with organ polarity, developmental transitions, auxin signaling, leaf and stem growth, floral organ identity, reproductive development and stress response [3–4, 7–12].

High-throughput sequencing combining with biological information analysis has improved the discovery of miRNAs in several plants due to the conservation of miRNAs among related plant species [13–21]. Recently, plenty of miRNA families have been discovered in plants [17–20]. With the application of degradome sequencing, miRNA targets in plants can be confirmed on a large scale [17–20]. Therefore, identification of miRNAs and their targets in diverse species have been a focus in recent years.

Maize (Zea mays L.), one of the most important crops in the world, is widely used as a model plant for biological research [22]. Over recent decades, several published reports about miRNAs in maize have focused on many biological processes, including leaf development, root development, seed development and response to stresses [23–31]. For instance, Juarez *et al*. showed miR166 constituted a highly conserved signal in maize leaf development [23]. Zhang *et al*. revealed that submergence-responsive miRNAs were involved in the regulation of metabolic, physiological and morphological adaptations of maize roots [24]. In addition, Kang *et al*. identified 125 and 127 known miRNAs from seeds and leaves in maize [25]. Ding *et al*. reported that 34 miRNAs belonging to 20 miRNA families were obtained in germinating maize seeds by high-throughput sequencing [26]. Furthermore, Li *et al*. used high-throughput sequencing to find that diverse and complex miRNAs were involved in the seed imbibition process [27]. Through degradome sequencing, Liu *et al*. detected that 72 genes targeted by 62 differentially expressed miRNAs might be attributed to the development of maize ears [28]. Jin *et al*. researched the dynamic expression patterns of miRNAs at 4 distinct developmental grain filling stages in maize [29]. Moreover, Wu *et al*. reported that miR811 and miR829 confer a high degree of resistance to *Exserohilum turcicum* [30]. Sheng *et al*. described the identification and characterization of novel miRNAs that are differentially expressed in drought-tolerant and drought-sensitive maize inbred lines [31].

At the early stage of the hybrid maize breeding in America, there were two major races, namely Southern Dent and Northern Flint [32]. After 1947, the maize races were divided into two major germplasm groups, namely Reid group (from Southern Dent) and Lancaster group (from Northern Flint) [32]. These two groups represent the main genetic diversity that is available for maize breeding in China and America [32–34]. In this study, miRNAs and their target transcripts in the PH6WC and PH4CV maize inbred lines, which were respectively from Reid and Lancaster groups [33–34], were investigated by using high-throughput sequencing and degradome analysis. The results indicated that some miRNAs (e.g. miR156, miR171, miR396 and miR444) differentially expressed in the seed development between PH6WC and PH4CV maize inbred lines under different genetic backgrounds.

## Materials and Methods

### Plant materials

Two maize inbred lines (i.e. PH6WC and PH4CV) were separately grown in experimental fields (JiangPu, Nanjing, China), without any artificial cultivation (for instance, fertilization and deinsectization). According to previous studies, the maximum value of the grain filling rate in maize seed development occurs between 21–25 days after pollination (DAP) [29, 35]. Therefore, samples form three stages (15, 25 and 45 DAP), which respectively represent early, medium and late stages in seed development, were selected and combined together for representing the whole process of the maize seed development in this study. In addition, all samples were frozen in liquid nitrogen and stored at -80°C. To minimize inter-individual differences, three samples from the same sampling location were mixed together.

### RNA extraction

Samples from PH6WC and PH4CV lines were individually subjected to RNA extraction using the TRIzol^®^ reagent (Invitrogen, Carlsbad, CA, USA). The quality of the extracted total RNAs was verified by using an Agilent 2100 Bioanalyzer (for concentration, 28S/18S and RIN detection; Agilent Technologies) and a NanoDrop 2000 spectrophotometer (for OD_260/280_ and OD_260/230_ detection; Thermo Fisher Scientific). In addition, the total RNAs were used for high-throughput sequencing, degradome analysis and qRT-PCR verification, identically.

### High-throughput sequencing and miRNA identification

Two small RNA (sRNA) libraries were constructed using the Illumina TruSeq Small RNA Preparation Kit (LC Sciences, Hangzhou, China). The total sRNAs were ligated to 3p and 5p adapters (ADTs), and the corresponding cDNA was obtained by reverse-transcription PCR. Following purification, the cDNA from the two sRNA libraries was sequenced using an Illumina HiSeq 2000 (LC Sciences, Hangzhou, China). Removing low-quality data, the raw reads were obtained using the Illumina Pipeline v1.5 (LC Sciences, Hangzhou, China). After removing ADTs, sequences with lengths <18 and >25 nt, junk data, mRNA fragments, Rfam and Repeats, the clean reads were subjected to miRNA identification by using the selected *Gramineae* pre-miRNAs/miRNAs database in miRBase 21.0 and the maize genome database. Three mismatches were allowed between the reads and the known pre-miRNAs/miRNAs sequences. As results, the reads that mapped to known pre-miRNAs/miRNAs and also mapped to the maize genome were identified as conserved miRNAs. In addition, the reads that did not map to known pre-miRNAs/miRNAs but mapped to the maize genome were considered as novel miRNAs. Furthermore, the secondary structures of all identified and potential pre-miRNAs in the maize genome were predicted by using the UNAFold software [36]. The minimal folding energy indexes (MFEIs) of the novel miRNAs should be equal or greater than 0.9 [37–39].

### Degradome sequencing and target identification

Two degradome libraries were constructed based on published methods [17, 19, 40]. Poly-A RNAs were obtained and ligated to a 5p adapter, and the cDNA was obtained by PCR. Following purification, the cDNA was sequenced through using an Illumina HiSeq 2000 (LC Sciences, Hangzhou, China). Removing low-quality data, the raw reads were obtained by using the Illumina Pipeline v1.5 (LC Sciences, Hangzhou, China). After removing ADTs and reads with lengths <15 nt, the remaining reads were compared with a cDNA library from the maize genome database. The mapped cDNA reads were then compared with the identified miRNAs to perform an alignment analysis by using CleaveLand 3.0 (LC Sciences, Hangzhou, China). The alignment scores ≤ 4 were considered. Furthermore, based on the number of degradome sequences and their abundance values, the miRNA targets were classified into 5 categories (0, 1, 2, 3 and 4, S5 Table) in accordance with reported method [17, 19, 40]. To further elucidate the potential functions, these miRNA targets were annotated through Gene Ontology (GO) and Kyoto Encyclopedia of Genes and Genomes (KEGG) [41].

### qRT-PCR

Total RNA extraction was performed and subsequently used for quantitative real-time PCR (qRT-PCR). qRT-PCR was conducted by using SYBR Premix Ex Taq^™^ (Takara, Dalian, China) system on a BIOER Line-Gene K RT-PCR machine (BIOER, Hangzhou, China). The primers were listed in S6.1 Table, and U6 was used as internal reference [42–43]. In addition, reactions were performed in triplicate, and relative expression levels were quantified by using 2^-ΔΔCt^ method (S6.2 Table).

### Statistical analysis

Log_2_(ratio) test and Chi-square 2×2 test were performed to identify differences in miRNA expression between the PH6WC and PH4CV libraries. Moreover, *p* values from Chi-square 2×2 test were adjusted to False Discovery Rate (FDR) as previous studies [44–45].

### Data availability

The datum of the high-throughput sequencing and degradome sequencing were deposited in the short read archive (SRA) in National Center of Biotechnology Information (NCBI). Their numbers are SRX1686992, SRX1686966, SRX1684509 and SRX1684462.

## Results

### Analysis of sRNA libraries

**Fig 1:**
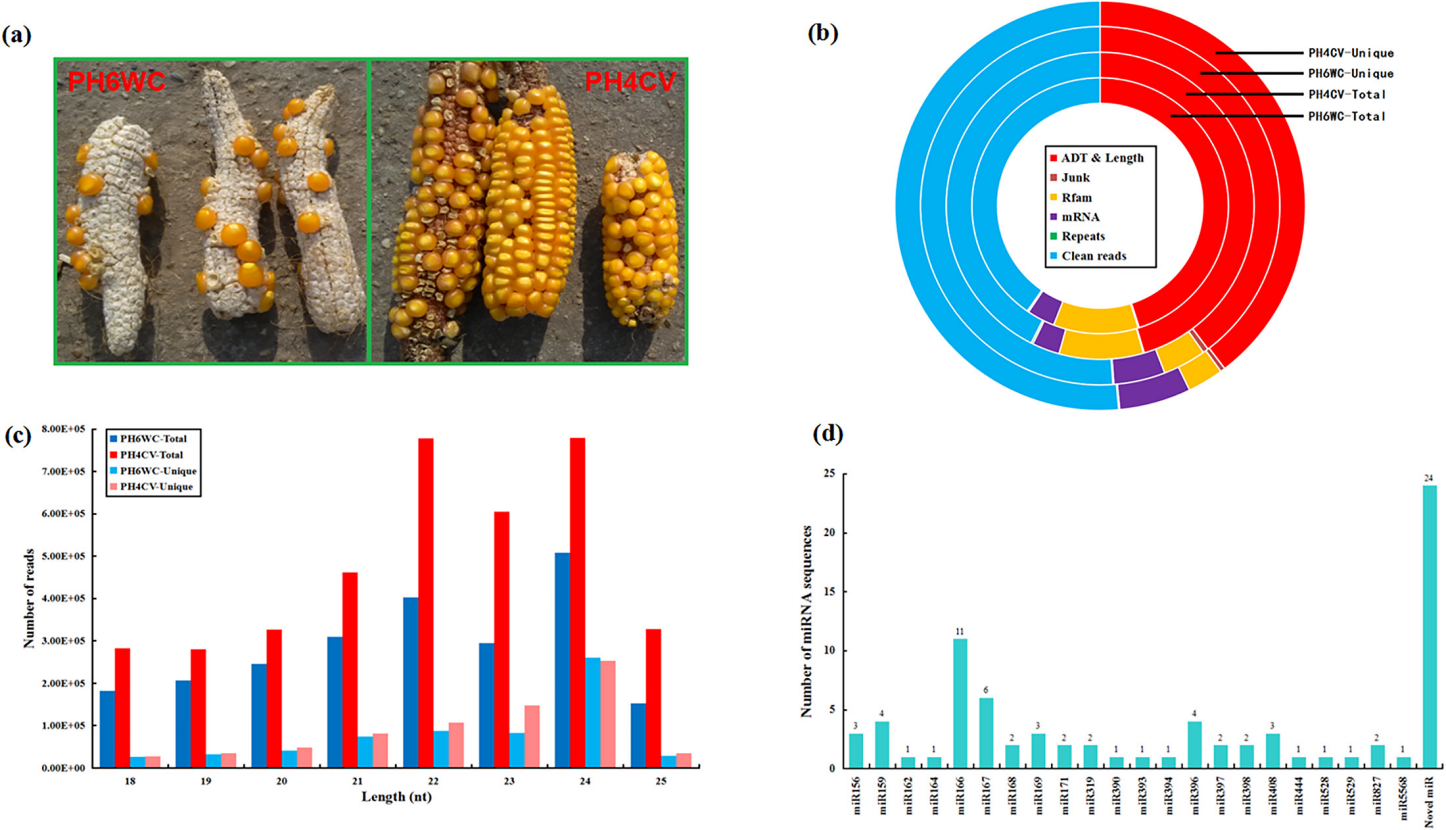
Summary of miRNA sequencing in the PH6WC and PH4CV libraries. (a) The mature maize cobs from the PH6WC and PH4CV lines; (b) Overview of sRNA sequences in the PH6WC and PH4CV libraries; (c) Length distributions of clean reads in the PH6WC and PH4CV libraries; (d) miRNA classification in the PH6WC and PH4CV libraries.

To identify miRNAs in seed develoment between two maize inbred lines (Fig 1a), two sRNA libraries were constructed. Following high-throughput sequencing, a total of 5,677,694 (from the PH6WC library) and 8,992,803 (from the PH4CV library) raw reads were generated (Fig 1b, S1 Table). After data filtering, 2,297,642 and 3,838,297 clean reads corresponding to 628,701 and 730,862 unique reads were obtained, respectively (Fig 1b, S1 Table). The distribution of the clean reads lengths was mainly between 21 and 24 nt (Fig 1c, S2 Table), which is consistent with previous reports [25–31].

### Identification of conserved and novel miRNAs

**Fig 2:**
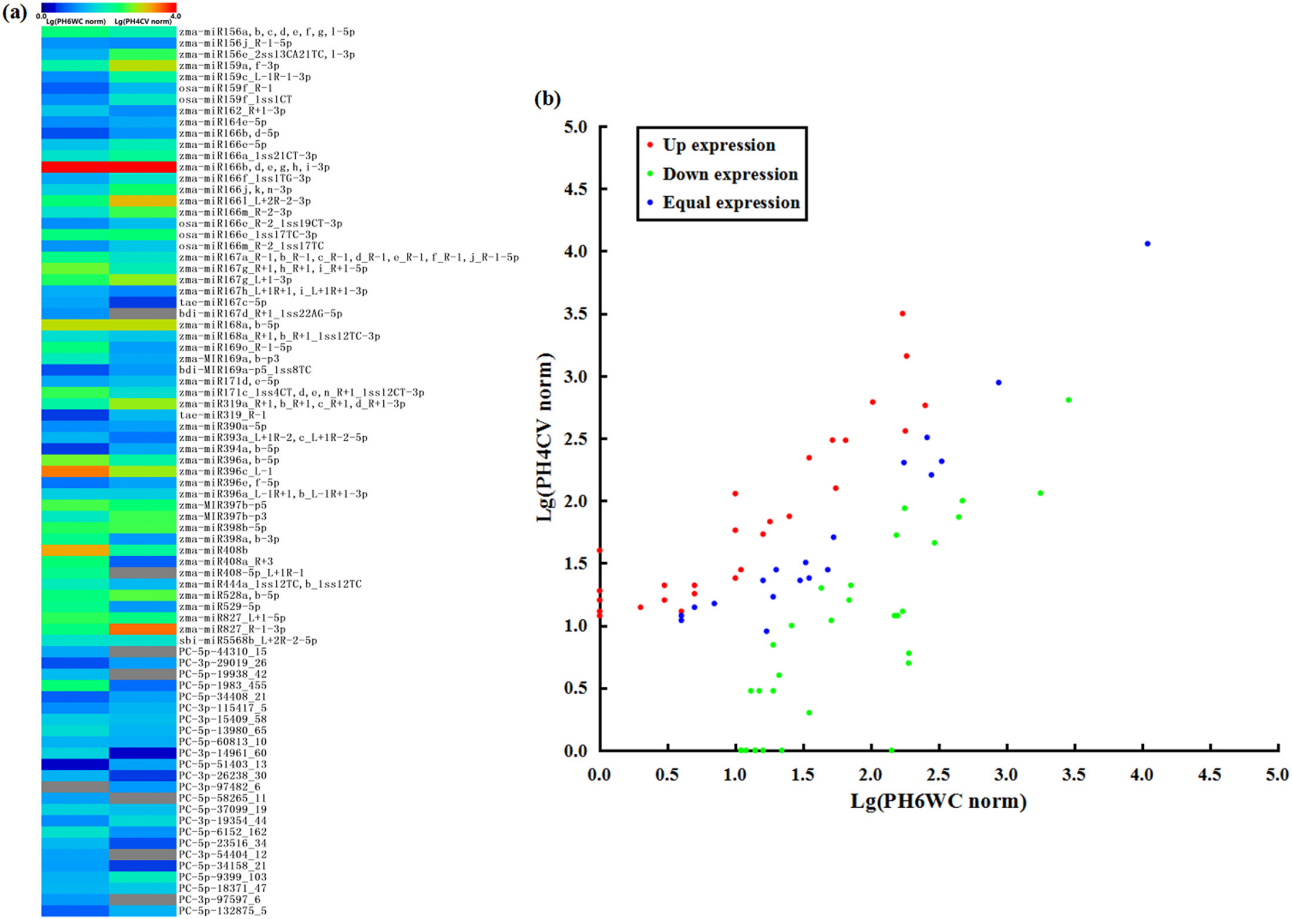
Expression comparison of miRNA sequences in the PH6WC and PH4CV libraries. (a) Overview of expression comparison of conserved and novel miRNAs in the PH6WC and PH4CV libraries. (b) Distributions of miRNA expression in the PH6WC and PH4CV libraries.

A total of 55 known unique miRNA sequences corresponding to 103 miRNAs were identified as conserved miRNAs, in which the miRNA sequences belonging to maize miRbase were identified as conserved known miRNAs (abbreviated as Con-K) and the sequences belonging to the selected *Gramineae* miRbase (except *Zea mays*) were identified as conserved novel miRNAs (abbreviated as Con-N) (S3 Table). Furthermore, these 55 known unique miRNA sequences were classified into 22 miRNA families (Fig 1d, S3 Table). On the other hand, a total of 24 novel unique miRNA sequences could form stem-loop structures with MFEI ≥ 0.9 were identified in two libraries (S3 Table). For expression comparison between the PH6WC and PH4CV libraries, unique miRNA sequences were analyzed through Log_2_(ratio) test and Chi-square 2×2 tests based on their normalized reads (Fig 2a, S3 Table). Following significant difference standard (*p* < 0.05 and |log_2_(PH4CV/PH6WC)| ≥ 1), 60 differentially expressed unique sequences were detected in two libraries (Fig 2, S3 Table). Comparing with the PH6WC library, 31 unique sequences were at up-expressed level and 29 unique sequences were at down-expressed level in the PH4CV library (Fig 2b, S3 Table).

### Identification of miRNA targets

The degradome analysis was performed in order to explore the potential miRNA targets. A total of 9,591,315 and 9,061,915 raw reads were generated from the PH6WC and PH4CV degradome libraries, respectively (S4 Table). After removing the ADTs and reads <15 nt, the remaining reads were compared with the *Zea mays* cDNA library. A total of 6,301,841 and 6,675,216 mapped cDNA reads were obtained from the two degradome libraries (S4 Table). The mapped cDNA reads were then compared with the identified miRNAs. Finally, a total of 137 miRNA targets corresponding to 38 unique miRNA sequences were discovered in the PH6WC and PH4CV degradome libraries (S5 Table). Among these, 74 targets were detected in both two libraries (S5 Table).

### Verification of miRNAs

**Fig 3:**
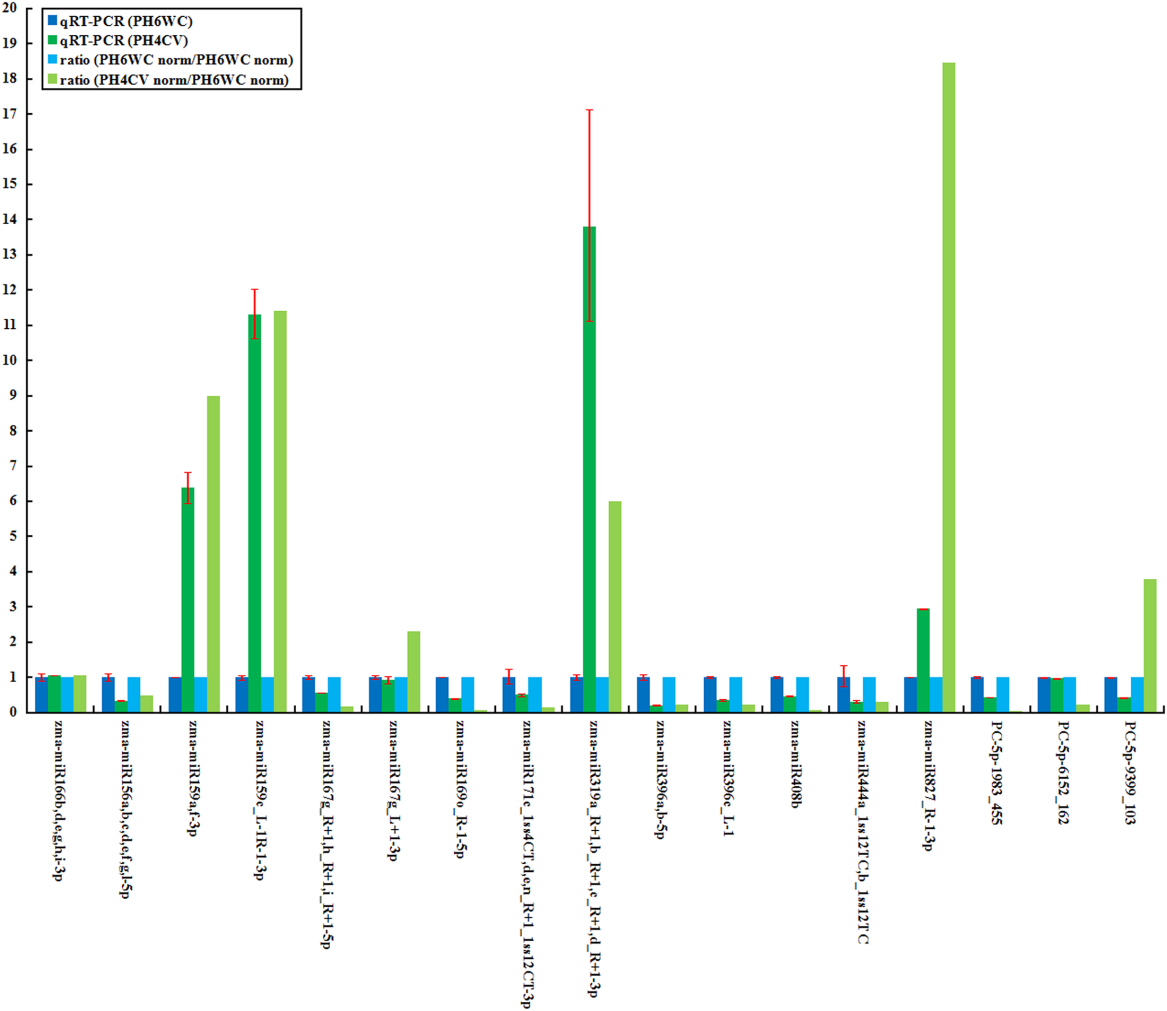
qRT-PCR validation of unique miRNA sequences in the PH6WC and PH4CV lines.

One extremely high expressed miRNA sequence (i.e. zma-miR166b,d,e,g,h,i-3p) and sixteen significantly differentially expressed miRNA sequences (normalized reads ≥ 50, *p* < 0.01 and |log_2_(PH4CV/PH6WC)| ≥ 1) were verified by qRT-PCR. The results nearly agreed with the high-throughput sequencing data (Fig 3, S3 Table). However, three of them were not in accordance with sequencing results. This presumably was due to differences in sensitivity and specificity from different approaches, which also appeared in previous studies [25–31].

## Discussion

High-throughput sequencing and degradome sequencing were used to identify differentially expressed miRNAs and their targets in seed development between PH6WC and PH4CV maize inbred lines. In total, 79 conserved and novel unique miRNA sequences corresponding to 127 miRNAs were identified in two inbred lines, in which 60 unique miRNA sequences existed different expression (*p* < 0.05 and |log_2_(PH4CV/PH6WC)| ≥ 1). Among these, 16 miRNA sequences showed significantly difference (normalized reads ≥ 50, *p* < 0.01 and |log2(PH4CV/PH6WC)| ≥ 1), and were verified by qRT-PCR.

Nine verified significantly differential miRNA sequences targeted 30 transcripts. By gene annotation, we found that most these targets were connected with maize reproductive development (Table 1). For instance, the targets of zma-miR156a,b,c,d,e,f,g,l-5p were predicted as *SPL10* and *SPL11* from Squamosa promoter-binding-like protein family, taking part in the regulation of timing of transition from vegetative to reproductive phase [46]. According to the previous studies, miR159 and miR319 probably from a homology family potentially regulate heterochronic development [47]. Similarly, zma-miR159c_L-1R-1-3p and zma-miR319a_R+1,b_R+1,c_R+1,d_R+1-3p in our study targeted to a same gene *TCP2*. Moreover, Gong *et al*. also reported that miR159 and miR319 might influence sweet corn seed vigor [48]. *APUM1* gene, might regulate mRNA translation [49], was considered as the target of zma-miR167g_R+1,h_R+1,i_R+1-5p. Additionally, *SCL6* gene was predicted as the target of zma-miR171c_1ss4CT,d,e,n_R+1_1ss12CT-3p. Previous studies declared *SCL* family is able to influence flowering time, which can control seed development [50–51]. In this present study, the major targets of zma-miR396a,b-5p and zma-miR396c_L-1 were *GRFs* (3, 5 and 6), members of growth-regulating factors, involved in regulation of cell expansion in leaf and cotyledons tissues [52]. This result agreed with previous study in miRNA identification in maize grain filling stages [29]. Besides, we found that *QQT2* was another target of zma-miR396a,b-5p. *QQT2* gene possibly is a regulator in embryo development [53]. *EDA18* gene as the target of zma-miR444a_1ss12TC,b_1ss12TC might participate in pollen and embryo sac development [54–55]. zma-miR827_R-1-3p should be conjectured to take part in maize seed development although its target was unknown in our study, because miR827 had obvious differences in expression level in our study and another previous study [29]. We believed that zma-miR166b,d,e,g,h,i-3p probably relate to the maize seed development, because 1) it high expressed in our study and other previous study [29]; 2) its target, *ATHB9* gene, is involved in the determination of adaxial-abaxial polarity in ovule primordium [56].

**Table 1:** qRT-PCR validated miRNAs and their targets in the PH6WC and PH4CV lines.

miRNA Name	Target Transcript	Transcript Annotation	Homologous Genes and Biological Function
zma-miR156a,b,c,d,e,f,g,l-5p	GRMZM2G126827_T01	SBP12; SBP transcription factor family protein	*SPL10*, *SPL11*; regulation of timing of transition from vegetative to reproductive phase [46]
	GRMZM2G156621_T01	SBP transcription factor family protein	*SPL10*, *SPL11*; regulation of timing of transition from vegetative to reproductive phase [46]
zma-miR159c_L-1R-1-3p	GRMZM2G020805_T01	TCP family transcription factor	*TCP2*; positive regulation of development, heterochronic [48]
zma-miR167g_R+1,h_R+1,i_R+1-5p	GRMZM2G042623_T01	Pumilio-family RNA binding protein	*APUM1*; regulation of translation [49]
	GRMZM2G042623_T02	Pumilio-family RNA binding protein	*APUM1*; regulation of translation [49]
zma-miR171c_1ss4CT,d,e,n_R+1_1ss12CT-3p	GRMZM2G037792_T01	GRAS79; GRAS transcription factor	*SCL6*; regulation in flowering time [50–51]
	GRMZM5G825321_T01	GRAS transcription factor	*SCL6*; regulation in flowering time [50–51]
	GRMZM5G825321_T02	GRAS transcription factor	*SCL6*; regulation in flowering time [50–51]
zma-miR319a_R+1,b_R+1,c_R+1,d_R+1-3p	GRMZM2G020805_T01	TCP family transcription factor	*TCP2*; positive regulation of development, heterochronic [48]
zma-miR396a,b-5p	GRMZM2G024293_T01	XPA-binding protein 1	*QQT2*; embryo development ending in seed dormancy [53]
	GRMZM2G024293_T03	XPA-binding protein 1	*QQT2*; embryo development ending in seed dormancy [53]
	GRMZM2G034876_T01	GRF transcription factor	*GRF5*; regulation of cell expansion in leaf and cotyledons tissues [52]
	GRMZM2G034876_T02	GRF transcription factor	*GRF5*; regulation of cell expansion in leaf and cotyledons tissues [52]
	GRMZM2G034876_T03	GRF transcription factor	*GRF5*; regulation of cell expansion in leaf and cotyledons tissues [52]
	GRMZM2G041223_T01	GRF6; GRF transcription factor	*GRF6*; regulation of cell expansion in leaf and cotyledons tissues [52]
	GRMZM2G045977_T01	GRF13; GRF transcription factor	*GRF3*; regulation of cell expansion in leaf and cotyledons tissues [52]
	GRMZM2G129147_T01	GRF transcription factor	*GRF5*; regulation of cell expansion in leaf and cotyledons tissues [52]
	GRMZM2G129147_T02	GRF transcription factor	*GRF5*; regulation of cell expansion in leaf and cotyledons tissues [52]
	GRMZM2G149543_T01	-	*-*
zma-miR396c_L-1	GRMZM2G034876_T01	GRF transcription factor	*GRF5*; regulation of cell expansion in leaf and cotyledons tissues [52]
	GRMZM2G034876_T02	GRF transcription factor	*GRF5*; regulation of cell expansion in leaf and cotyledons tissues [52]
	GRMZM2G034876_T03	GRF transcription factor	*GRF5*; regulation of cell expansion in leaf and cotyledons tissues [52]
	GRMZM2G041223_T01	GRF6; GRF transcription factor	*GRF6*; regulation of cell expansion in leaf and cotyledons tissues [52]
	GRMZM2G045977_T01	GRF13; GRF transcription factor	*GRF3*; regulation of cell expansion in leaf and cotyledons tissues [52]
	GRMZM2G129147_T01	GRF transcription factor	*GRF5*; regulation of cell expansion in leaf and cotyledons tissues [52]
	GRMZM2G129147_T02	GRF transcription factor	*GRF5*; regulation of cell expansion in leaf and cotyledons tissues [52]
zma-miR444a_1ss12TC,b_1ss12TC	GRMZM2G001024_T01	RING/U-box superfamily protein	*EDA18;* pollen and embryo sac development [54–55]
	GRMZM2G001024_T02	RING/U-box superfamily protein	*EDA18;* pollen and embryo sac development [54–55]
	GRMZM2G001024_T03	RING/U-box superfamily protein	*EDA18;* pollen and embryo sac development [54–55]
zma-miR827_R-1-3p	GRMZM2G003992_T01	-	*-*
zma-miR166b,d,e,g,h,i-3p	GRMZM2G038198_T01	START domain containing protein	*ATHB9*; determination of adaxial-abaxial polarity in ovule primordium [56]

**Fig 4:**
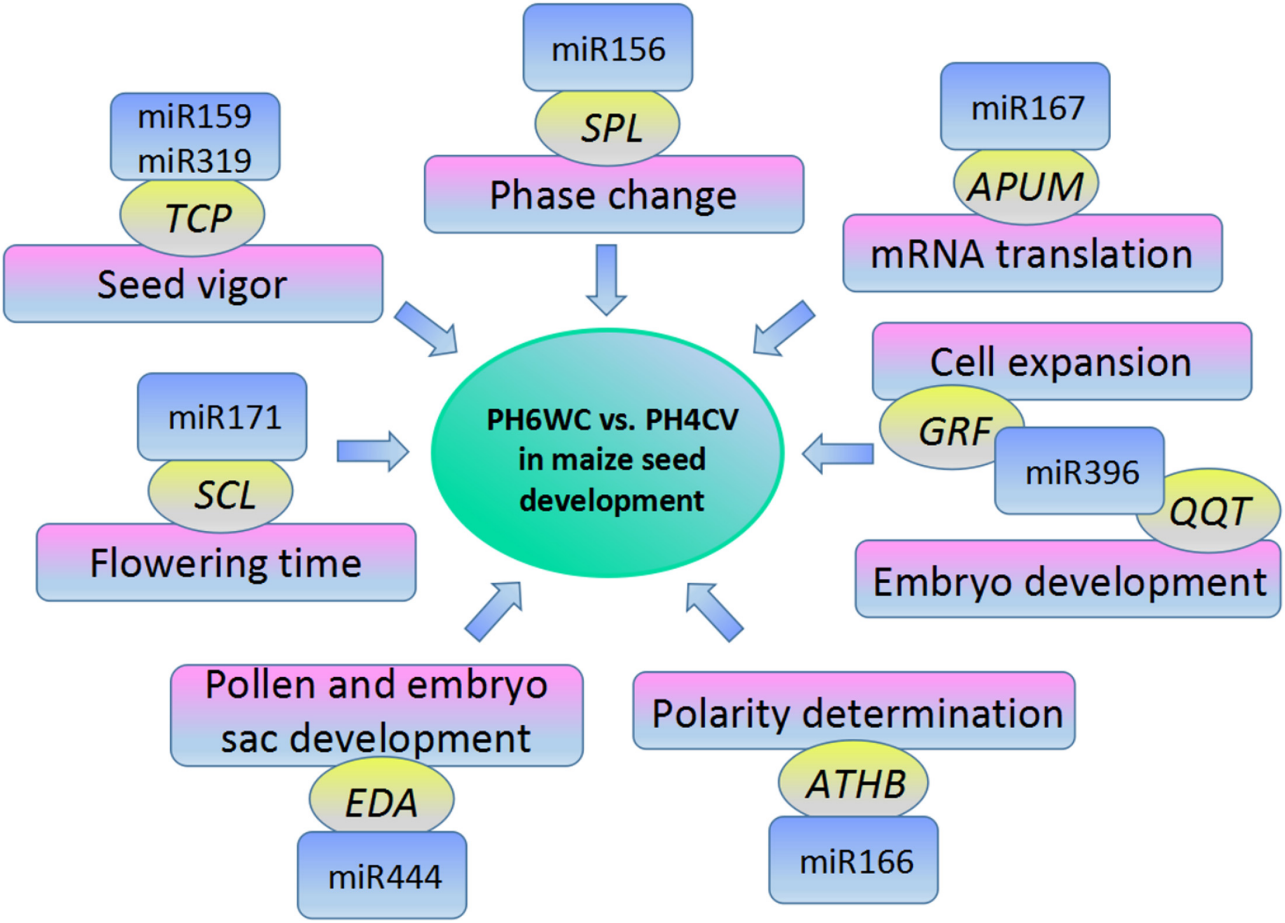
Differentially expressed miRNAs and their targets involved in seed development between the PH6WC and PH4CV lines.

In summary, our preliminary results suggested some miRNAs differentially expressed in seed development between PH6WC and PH4CV inbred lines. This situation we found might be an evidence that can prove the complexity of the maize genetic background on miRNAs level. As reported in previous studies, two maize lines might be on average as diverged from one another as humans are from chimpanzees [57]. Furthermore, the targets of the miRNAs differentially expressed in this study mainly involved with regulation in flowering (e.g. *SPL* and *SCL*) and development in embryo (*QQT* and *EDA*), and might have coordinated functions in the maize seed development (Fig 4).

## Conclusions

In this study, several differentially expressed miRNAs and their targets were identified in seed development in two maize inbred lines (i.e. PH4CV and PH6WC) by using high-throughput sequencing and degradome analysis. The results indicated that miR156, miR171, miR396 and miR444, which respectively targeted to *SPL*, *SCL*, *QQT* and *EDA* genes, might differentially expressed in the seed development in two maize inbred lines, especially involved in flowering regulation and embryo development. Therefore, this preliminary results might improve our understanding on the regulatory roles of miRNAs in maize seed development under different genetic backgrounds.

## Supporting Information

S1 Table Overview of sRNA sequences in the PH6WC and PH4CV libraries. (XLSX)

S2 Table Length distributions of clean reads in the PH6WC and PH4CV libraries. (XLSX)

S3 Table Conserved and novel miRNAs in the PH6WC and PH4CV libraries. (XLSX)

S4 Table Overview of data in the PH6WC and PH4CV degradome libraries. (XLSX)

S5 Table Targets of identified miRNAs in the PH6WC and PH4CV degradome libraries. (XLSX)

S6 Table qRT-PCR verification and primers of miRNAs in the PH6WC and PH4CV lines. (XLSX)
